# Optimization of β-Fructofuranosidase Production from Agrowaste by *Aspergillus carbonarius* and Its Application in the Production of Inverted Sugar

**DOI:** 10.17113/ftb.59.03.21.6934

**Published:** 2021-09

**Authors:** Ryhára Dias Batista, Fernanda Guimarães Melo, Claudia Cristina Auler do Amaral Santos, Fabrício Coutinho de Paula-Elias, Rafael Firmani Perna, Michelle Cunha Abreu Xavier, Sergio Andres Villalba Morales, Alex Fernando de Almeida

**Affiliations:** 1 Graduate Program in Food Science and Technology, Federal University of Tocantins, 109 Norte Av. NS-15, ALCNO-14, Plano Diretor Norte, CEP: 77001-090, Palmas, Tocantins, Brazil; 2 Federal University of Alfenas (UNIFAL-MG), Institute of Science and Technology, José Aurélio Vilela Road 11999, Km 533, Zip Code 37715-400, Poços de Caldas, MG, Brazil; 3 Federal University of Tocantins (UFT), Department of Bioprocess Engineering and Biotechnology, Badejos Street 69-72, Jardim Cervilha, Zip Code 77404-970, Gurupi, TO, Brazil

**Keywords:** invertase, *Aspergillus carbonarius* PC-4, culture optimization, simplex lattice design, sucrose hydrolysis

## Abstract

**Research background:**

Microbial β-fructofuranosidases are widely employed in food industry to produce inverted sugar or fructooligosaccharides. In this study, a newly isolated *Aspergillus carbonarius* PC-4 strain was used to optimize the β-fructofuranosidase production in a cost-effective process and the sucrose hydrolysis was evaluated to produce inverted sugars.

**Experimental approach:**

Optimization of nutritional components of culture medium was carried out using simplex lattice mixture design for 72 and 120 h at 28 °C. One-factor-at-a-time methodology was used to optimize the physicochemical parameters. Crude enzyme was used for sucrose hydrolysis at different concentrations.

**Results and conclusions:**

The optimized conditions of enzyme production were achieved from cultivations containing pineapple crown waste (1.3%, *m/V*) and yeast extract (0.3%, *m/V*) after 72 h with an enzyme activity of 9.4 U/mL, obtaining R^2^=91.85%, R^2^_adjusted_=85.06%, highest *F*-value (13.52) and low p-value (0.003). One-factor-at-a-time used for optimizing the physicochemical conditions showed optimum temperature (20 °C), pH (5.5), agitation (180 rpm) and time course (72 h) with a 3-fold increase of enzyme production. The invertase-induced sucrose hydrolysis showed the maximum yield (3.45 mmol of reducing sugars) using 10% of initial sucrose concentration. Higher sucrose concentrations caused the inhibition of invertase activity, possibly due to the saturation of substrate or formation of sucrose aggregates, making it difficult for the enzyme to access sucrose molecules within the created clusters. Therefore, a cost-effective method was developed for the invertase production using agroindustrial waste and the produced enzyme can be used efficiently for inverted sugar production at high sucrose concentration.

**Novelty and scientific contribution:**

This study presents an efficient utilization of pineapple crown waste to produce invertase by a newly isolated *Aspergillus carbonarius* PC-4 strain. This enzyme exhibited a good potential for inverted sugar production at high initial sucrose concentration, which is interesting for industrial applications.

## INTRODUCTION

Invertases (β-d-fructofuranoside fructohydrolases, E.C. 3.2.1.26) are glycoside hydrolases that catalyze the hydrolysis of sucrose into d-glucose and d-fructose. These enzymes naturally occur in plant, bacteria, yeasts and filamentous fungi (*1*, *2*). Invertases have drawn the attention of different food and beverage industries due to their feature of generating equimolar mixtures of glucose and fructose, which is the basis to produce high fructose syrups (*3*). The sucrose hydrolysis by invertases produces a mixture of sugars called inverted sugar since sucrose rotates its plane-polarized light to the right (dextrorotatory), whereas the hydrolysis products deviate the plane-polarized light to the left (levorotatory) (*4*). Inverted syrup with high fructose concentration is used in diverse food industries such as jam and jellied products. It minimizes the crystallization rate and maintains softness of sweetmeats, so the products will remain soft throughout their shelf life (*5*). Therefore, invertases have been associated with the industry of bakery and beverages, reducing 5-15% of sugar content of soft drinks with the same sweetness. Moreover, invertases can exhibit transfructosylation activity for production of fructooligosaccharides at higher sucrose concentrations with high nutraceutical properties (prebiotics) (*2*).

A diversity of cultivation strategies based principally on submerged and solid-state fermentation has been used to produce fungal invertases. For industrial microbial cultivation process, medium composition plays a critical role due to its major influence on cell growth and microbial physiology leading to the formation of products (*6*). Optimization of cultivation medium is one of the critical stages of industrial production and must be carried out before scaling up the synthesis of microbial metabolites. Therefore, the optimized production and the variables that affect the enzymatic production should always be investigated, as the optimal conditions vary according to different microbial strains and their respective enzyme synthesis (*7*). For each bioproduct, an increase in productivity reduces the overall cost of the product. Hence, it is one of the most important topics for enzyme research (*6*, *8*).

Agroindustrial wastes are utilized in many bioprocesses due to particular interest in renewability, low cost, and suitable characteristics which allow the production of different value-added metabolites. The state of Tocantins, Brazil, produces 54 million pineapple fruits annually. Pineapple products are classified into two categories: pineapple crown as a postharvest waste, and waste from fruit processing industry. Pineapple crown waste is an important and available source of lignocellulosic biomass and its disposal can cause significant environmental problems. The pineapple waste appears to be a valuable substrate with great potential if appropriate processes and technologies are applied to transform its different components (*4*). The aim of this work is to optimize the cultivation parameters for invertase production by *Aspergillus carbonarius* PC-4 using pineapple crown waste as a substrate, a simplex lattice design model for quantification of nutritional components and one-factor-at-a-time method for determination of physicochemical parameters of cultivation, apart from studying the biocatalyst application for sucrose hydrolysis at different concentrations to synthesize glucose and fructose as reducing sugars.

## MATERIALS AND METHODS

### Biocatalyst and microbial cultivations

*Aspergillus carbonarius* PC-4 was isolated from canned peach syrup and it is available in the Laboratory of Biotechnology and Analysis of Food and Products, Federal University of Tocantins, Gurupi, Tocantins, Brazil. The strain was identified by morphological and molecular techniques (*9*). The gene sequence of *A. carbonarius* PC-4 was deposited in the NCBI Genbank database and it is available under the accession number AJ876878. Spore suspension was transferred to Petri plates containing potato dextrose agar (PDA; Difco, St Louis, MO, USA) and incubated at 30 °C for five days. Isolated colonies were inoculated in the center of Petri dishes to ensure the purity of fungal strains. Samples of this strain with a portion of the solid culture medium (5 mm×10 mm) were transferred to sterile glass flasks (6 mL) and filled with 4 mL sterile distilled water, which were labelled and hermetically closed with rubber stoppers and aluminium seals (*10*). Castellani bottles were kept at 4 °C and the viability of strains was verified periodically. The filamentous fungal strain was streaked on PDA slant agar and incubated at 28 °C for 72 h prior to shake flask experiments.

Vogel’s medium was used for enzyme production (*11*). Trace element solution (solution A) was prepared containing (g/L): C_6_H_8_O_7_∙H_2_O 50, ZnSO_4_·7H_2_O 50, Fe(NH_4_)_2_(SO_4_)_2_·6H_2_O 10, CuSO_4_·5H_2_O 2.5, MnSO_4_·H_2_O 0.05, H_3_BO_3_ 0.05 and Na_2_MoO_4_·2H_2_O 0.05. Salt solution (solution B) was prepared containing (g/L): Na_3_C_6_H_5_O_7_·5H_2_O 150, KH_2_PO_4_ 250, NH_4_NO_3_ 100, MgSO_4_·7H_2_O 10 and CaCl_2_·2H_2_O 5. To 1 L of solution B, 5 mL of biotin (0.1 mg/mL), 5 mL of solution A and 0.2 mL of chloroform were added. All solutions were stored at 4 °C. Medium preparation consisted of 50-fold dilution of solution B. Inoculum was obtained from PDA slant agar cultivations by preparing conidial suspensions with distilled water and adjusted to 10^6^ spore/mL. A volume of 1 mL was used to inoculate 20 mL Vogel’s salt medium at pH=6 in Erlenmeyer flasks (125 mL), which was supplemented with pineapple crown as carbon source, besides ammonium chloride and yeast extract as nitrogen sources. Pineapple crown waste was obtained from producers from Miracema do Tocantins, Tocantins, Brazil. It was initially dried and ground to a powder (30 mesh). Then, this powder was washed extensively to remove free soluble sugars. All cultivation media were sterilized at 121 °C for 15 min (Vertical Autoclave CS-50, Prismatec, São Paulo, Brazil). Shake flasks were incubated at 28 °C and 180 rpm for 72-120 h. After cultivation, biomass was separated by vacuum filtration system (Solab, São Paulo, Brazil) and determined gravimetrically, which was expressed in gram per liter of culture medium.

### Physicochemical conditions for enzyme production

The influence of temperature on invertase production was evaluated by varying the temperature from 15 to 45 °C, with intervals of 5 °C. This first experimental set was performed for 72 h at 180 rpm in an orbital shaker (Lucadema, São Paulo, Brazil), pH=6.0. The second set of experiments was conducted to evaluate the influence of different initial pH values on invertase production. The initial medium pH was adjusted by the addition of 1 mol/L NaOH or HCl with values ranging from 3.0 to 8.0. These cultivations were also performed for 72 h at 180 rpm and at an optimum temperature of 20 °C. The third experimental set was accomplished aiming at the analysis of different agitation rates: 130, 150, 180 and 210 rpm. These experiments were conducted at 20 °C and initial pH=5.5 for 120 h. Samples from all experimental sets were taken at intervals of 12 h. Cell-free broth was used for enzyme and protein assays.

Invertase activity was determined by adding 0.2 mL crude enzyme to 0.8 mL substrate solution containing 2% (*m*/*V*) sucrose diluted in 0.1 mol/L citrate-phosphate buffer, pH=5.0. Enzyme assays were performed at 50 °C for 5 min. Samples of 0.2 mL from the enzyme reaction solution were transferred to reaction tubes with 0.2 mL of the reagent 3,5-dinitrosalisylic acid and heated at 100 °C for 5 min in a water bath (Cienlab, São Paulo, Brazil), according to Miller (*12*). After that, a volume of 2 mL of distilled water was added and the absorbance of samples was measured at 540 nm using a spectrophotometer UV/Vis UV 380G (Gehaka, São Paulo, Brazil). Glucose solution (1 g/L) was used for the standard curve. One invertase unit was defined as the amount of enzyme that released 1 µmol of reducing sugar per min under the aforementioned conditions. Invertase production yield was calculated by dividing enzyme units per gram of consumed carbon source, whereas invertase productivity was obtained as the amount of enzyme units per h of cultivation. Protein was determined according to Lowry *et al*. (*13*), using bovine serum albumin as standard. All experiments were carried out in triplicate.

### Sucrose hydrolysis

Sucrose solutions of 1, 5, 10, 15, 20 and 30% (*m/V*) in 0.1 mol/L citrate-phosphate buffer (pH=5.0) were submitted to enzyme hydrolysis. A volume of 10 mL sucrose solution was maintained for 5 min at 50 °C with subsequent addition of 0.1 mL crude enzyme to the reaction tubes, and then sucrose hydrolysis was performed for 180 min. Samples of 0.4 mL were taken at different intervals and subjected to enzyme inactivation by boiling at 100 °C for 5 min, then 0.2 mL were added to 3,5-dinitrosalicylic acid to determine the amount of reducing sugars produced after hydrolysis reaction. Results were expressed in µmol of reducing sugar. All experiments were carried out in triplicate.

### Experimental design

A simplex lattice mixture design was performed for culture medium optimization for invertase production. The model was fitted to evaluate the influence of three factors on invertase production: ammonium chloride (X_1_), yeast extract (X_2_) and pineapple crown (X_3_). The experimental set considered three levels of concentration for each input variable (0, 0.5 and 1). The experimental design totalized 12 experiments, which were performed in triplicates. The data factors were chosen after a series of preliminary assays (data not shown). Table 1 shows the experimental points of mixture design with respective real values of variable levels.

**Table 1:** Simplex lattice mixture design arrangement of the actual and coded independent variables for β-fructofuranosidase production by Aspergillus carbonarius PC-4

	Ammonium chloride	*γ*/(g/L) Yeast extract	Pineapple crown	*t*/h						
Run		72				120	
	Invertase activity/ (U/mL)	*Y*_p/s_/(U/g)	*r*_p_/(U/h)		Invertase activity/(U/mL)	*Y*_p/s_/(U/g)	*r*_p_/(U/h)
1	10.0 (1.0)	0.0 (0.0)	1.0 (0.0)	1.4±0.1	1404.3	0.4		3.0±0.0	2431.0	0.4
2	0.0 (0.0)	10.0 (1.0)	1.0 (0.0)	3.9±0.0	3121.6	0.9		5.6±0.1	4803.8	0.8
3	0.0 (0.0)	0.0 (0.0)	20.0 (1.0)	9.0±0.2	361.9	2.0		9.2±0,1	346.2	1.1
4	5.0 (0.5)	5.0 (0.5)	1.0 (0.0)	4.3±0.0	3884.7	1.1		5.9±0.0	5345.4	0.9
5	5.0 (0.5)	0.0 (0.0)	10.0 (0.5)	9.7±0.2	775.3	2.1		9.6±0.1	866.0	1.4
6	0.0 (0.0)	5.0 (0.5)	10.0 (0.5)	8.9±0.0	669.9	1.9		9.8±0.2	833.6	1.4
7	10.0 (1.0)	0.0 (0.0)	1.0 (0.0)	1.0±0.1	1047.4	0.3		2.0±0.1	1802.5	0.3
8	0.0 (0.0)	10.0 (1.0)	1.0 (0.0)	2.8±0.2	2210.0	0.6		3.9±0.1	3491.1	0.6
9	0.0 (0.0)	0.0 (0.0)	20.0 (1.0)	7.5±0.2	281.4	1.6		10.0±0.1	357.7	1.2
10	5.0 (0.5)	5.0 (0.5)	1.0 (0.0)	4.8±0.0	4079.8	1.1		6.3±0.1	5676.9	0.9
11	5.0 (0.5)	0.0 (0.0)	10.0 (0.5)	6.0±0.2	480.6	1.3		6.1±0.3	630.8	1.0
12	0.0 (0.0)	5.0 (0.5)	10.0 (0.5)	9.6±0.2	723.4	2.0		8.4±0.2	716.2	1.2

*Y*_p/s_=yield of invertase, *r*_p_=productivity of invertase

### Statistical analysis

The statistical analysis of the data was carried out using STATISTICA software, v. 13.4 (*14*). The significant level was p<0.05. The quadratic regression analysis was performed for all response variables in this work, as follows:


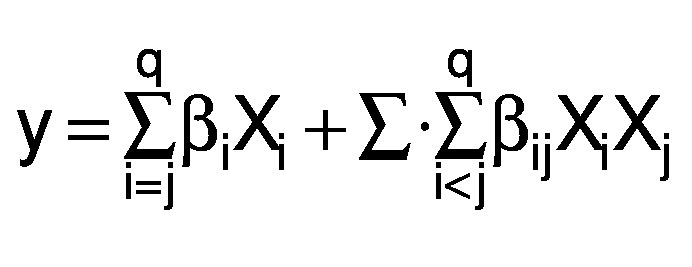


where y is the response variable and corresponds to invertase activity (U/mL), q is the number of ingredients for the design experiment, X_i_ and X_j_ are proportion variables, β_i_ is the regression coefficient for linear terms, whereas β_ij_ refers to quadratic terms of the model with binary interaction. Additional experiments were performed to validate the obtained model with optimized values of component variables.

For the analysis of physicochemical conditions for invertase production, the data were subjected to analysis of variance (one-way ANOVA) by BioEstat 5.0 (*15*), and Tukey’s test with p≤0.05 was used for the evaluation of statistically significant differences.

## RESULTS AND DISCUSSION

### Mixture design experiment

Preliminary experiments with *Aspergillus carbonarius* PC-4 showed an invertase activity of 6.7 U/mL with a production yield of 587 U/g of substrate and a productivity of 1.6 U/h (*9*). Furthermore, the mixture design was chosen to analyze the effect of relative ratios of the ingredients on the composition of the medium, since the main distinction between this experimental design and independent variable experiments is that the input variables are non-negative proportionate amounts in the mixture, which are expressed as fractions of total amount of the mixture. Ammonium chloride was also tested as a component of the simplex lattice mixture design besides pineapple crown and yeast extract in the respective levels of the performed experimental design.

**Fig. 1:**
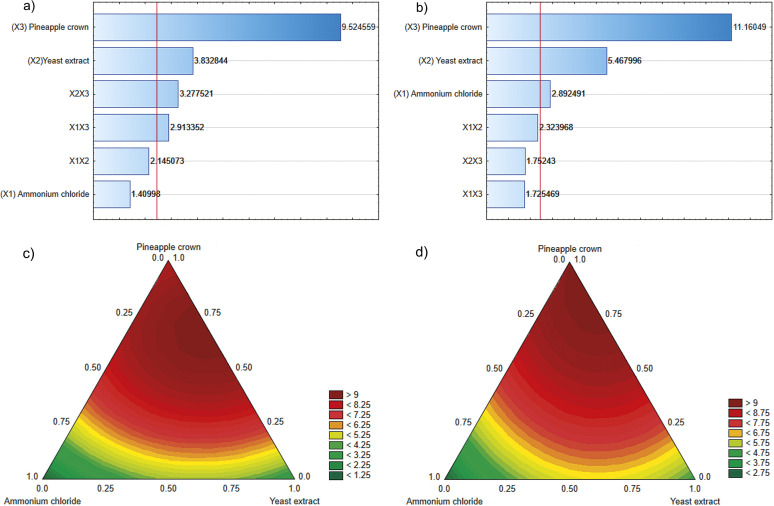
Pareto charts and quadratic contour area of ternary plots of the effect of culture medium variables on β-fructofuranosidase activity (U/mL) by *Aspergillus carbonarius* PC-4 after: a and c) 72 h and b and d) 120 h (significance p<0.05)

Table 1 shows invertase activities, invertase production yield (*Y*_p/s_) and enzyme productivity (*r*_p_) for varying compositions of substrates in each experimental run. The invertase activity ranged from 1.0 to 9.7 U/mL during cultivation for 72 h and from 2.0 to 10.0 U/mL during cultivation for 120 h, which showed the effect of component variables on fermentative parameters for enzyme production. Analyses based on *t*-test and p-value (<0.05) for invertase production as the response showed that yeast extract and pineapple crown were significant ingredients of the mixture for cultivation for 72 and 120 h, whereas ammonium chloride was significant only after 120 h of cultivation. The effect of evaluated factors on enzyme activity is given in the Pareto charts (Figs. 1a and 1b).

The ternary plots show models obtained after 72 and 120 h of cultivation, indicating the existence of optimal region for invertase activity located near the top of the design triangle for both periods of cultivation (Figs. 1c and 1d). Possibly, higher concentrations of pineapple crown and intermediate concentrations of yeast extract as carbon and nitrogen sources, respectively, significantly influenced the predicted values of enzymatic activity (above 9 U/mL). Silva *et al*. (*16*) showed that pineapple crown is a potential carbon source, containing xylose and mostly glucose as constituents, and hence is an attractive residue for microbial growth. The predicted *versus* observed values revealed a good correlation between the model and experimental invertase production. The simplex lattice design methodology promoted a 1.42-fold increase of enzyme production, when compared to the results obtained by Nascimento *et al*. (*9*). Under optimized culture conditions, the production parameters were 723.4 U/g for production yield and 2 U/h for enzyme productivity.

ANOVA results for quadratic model showed an R^2^ value of 91.85% and an adjusted R^2^ value of 85.06% after 72 h of cultivation. On the other hand, the analysis of variance of cultivation for 120 h revealed an R^2^ value of 89.33% and an adjusted R^2^ of 80.43% (Table 2). From these two models, 72-hour cultivation was chosen for further experiments, whose substrate components would explain 91.85% of variability in the response variable, leaving 8.15% of the variability unexplained. The quadratic model for 72-hour cultivation showed a significant *F*-value (13.52) and a low p-value (0.003), which is an interesting result since a shorter period of cultivation with similar enzyme activity values resulted in better enzyme production yields. The obtained second order models express the empirical relationship between the invertase production and the ingredients of the mixture (ammonium chloride, yeast extract and pineapple crown) during 72 and 120 h of cultivation, respectively:


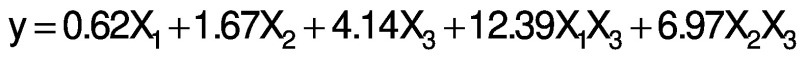



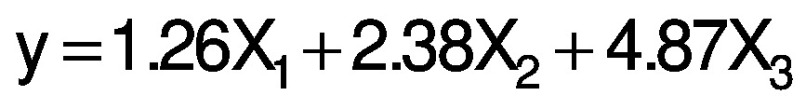


**Table 2:** ANOVA for significance of the regression models

Time/h																	
72								120									
Source	Sum of squares	DF	Mean square	*F*-value	p-value				Sum of squares		DF		Mean square		*F*-value		p-value
Model	102.09	5	20.42	13.52	0.003			76.28		5		15.25		10.04		0.007	
Total error	9.06	6	1.51					9.11		6		1.52					
Lack of fit	0.00	0	0.00					0.00		0		0.00					
Pure error	9.05	6	1.51					9.11		6		1.52					
Total adjusted	111.15	11	10.10					85.39		11		7.76					
Factor	Effect	Error	*t*-value	p-value				Factor		Effect		Error		*t*-value		p-value	
Ammonium chloride (X_1_)	1.22	0.87	1.41	0.21				2.52		0.87		2.89		0.03		2.52	
Yeast extract (X_2_)	3.33	0.87	3.83	0.01				4.76		0.87		5.47		0.00		4.76	
X_1_∙X_2_	8.27	0.87	9.52	0.00				9.73		0.87		11.16		0.00		9.73	
X_1_∙X_3_	9.12	4.26	2.14	0.07				9.92		4.27		2.32		0.06		9.92	
X_2_∙X_3_	12.40	4.26	2.91	0.03				7.37		4.27		1.72		0.13		7.37	
X_1_∙X_2_	13.94	4.26	3.27	0.02				7.48		4.27		1.75		0.13		7.48	

For fermentation time of 72 and 120 h R^2^=91.85%, R^2^_adjusted_=85.06%, and R^2^=89.33%, R^2^_adjusted_=80.43%, respectively. DF=degree of freedom, X_3_=pineapple crown

where y is invertase activity (U/mL), X_1_ is ammonium chloride, X_2_ is yeast extract, and X_3_ is pineapple crown concentrations.

Chemical characterization of pineapple crown waste showed that it contained 28.6% total carbon, 1.8% total nitrogen, with a C/N ratio of 15.7, 7.7% humidity, 6.0% ash, 6.7% total lipids, 11.5% total protein and 68.1% total carbohydrates. Therefore, in this study, an efficient utilization of pineapple crown waste by *A. carbonarius* PC-4 to produce invertase under submerged culture condition was observed. The invertase production by *Aspergillus* strains isolated from agroindustrial residues and by-products has been extensively studied in the last decades. These substrates are rich in nutrients such as carbon and nitrogen sources for the microbial growth and they are also inexpensive, which is economically interesting (*17*, *18*). Oyedeji *et al*. (*18*) observed the increase of invertase production by *A. niger* IBK1 due to utilization of pineapple peel for growth and hence enzyme production. Pineapple peel contains a considerable amount of soluble sugars such as sucrose, which makes it suitable for use as a substrate in microbial fermentations. Thus, the use of pineapple peel for fungal growth is attractive, since it is inexpensive and rich in carbon and nitrogen and also a source of minerals (*19*).

### Validation of the experimental model

The results predicted by the second order model developed for invertase production by *A. carbonarius* PC-4 suggested an optimal production with medium supplemented with 13.5 g/L pineapple crown and 3.5 g/L yeast extract (invertase activity=((9.7±0.4) U/mL). The validation experiments corroborate the model and show an invertase production of similar value ((9.4±0.4) U/mL) suggested by the second order model (data not shown).

### Influence of temperature, pH and agitation speed on cell growth and enzyme synthesis

**Fig. S1:**
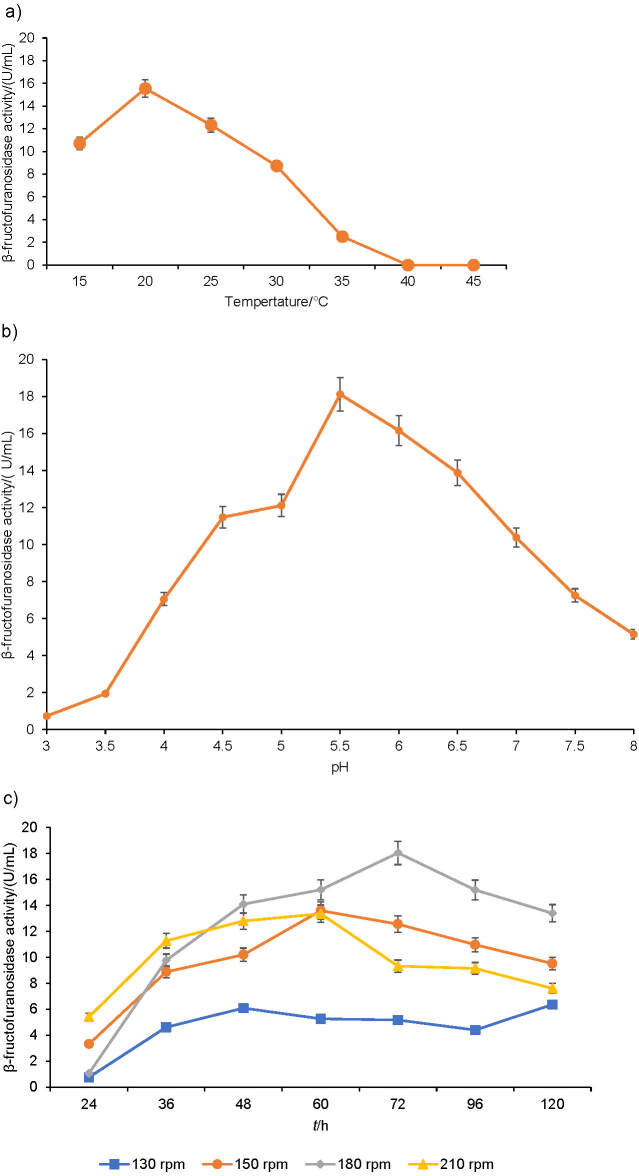
Effect of: a) temperature, b) pH and c) agitation speed on β-fructofuranosidase production by *Aspergillus carbonarius* PC-4 under submerged conditions. Culture conditions: effect of temperature and pH was measured for 72 h, while agitation speed experiments were carried out under optimized conditions for 120 h of cultivation

The effect of temperature, initial pH and agitation speed on enzyme activity can be observed in Fig. S1. The cultivation temperatures ranging from 15 to 45 °C were used to determine their effects on cell growth and invertase production by *A. carbonarius* PC-4. Additional culture conditions were previously established and maintained (120 h cultivation, 180 rpm and initial pH=6.0) (Fig. S1a). The increase of incubation temperature to 20 °C resulted in the highest invertase production ((15.5±0.8) U/mL), with yield and productivity values of 783.2 U/g and 2.92 U/h, respectively (Table S1). Invertase production and fermentation parameters remained at high levels until 30 °C, with decreasing values observed above this temperature. Enzyme production was not observed at 40 and 45 °C. Other enzymes, *e.g*. pectinases, amylases, glucosidases and proteases were produced by *A. carbonarius* strains at cultivation temperatures 28-30 °C (*20*-*23*). The effect of temperature on invertase production by filamentous fungi was widely reported in the literature (*3*, *10*, *18*). *Aspergillus* spp. strains are generally grown at 30 °C for invertase production. In this work, the growth temperature of 20 °C for *A. carbonarius* PC-4 can be explained by the fact that this strain has been isolated from canned peach syrup maintained at low temperature.

The initial pH of cultivation medium is an important physical parameter that affects microbial growth, metabolic activity maintenance and enzyme production by *Aspergillus* spp. strains (*18*, *24*, *25*). Cultivation at different initial pH values was used to evaluate the invertase production by *A. carbonarius* PC-4, maintaining the conditions previously established (120 h, 180 rpm and 20 °C). The invertase production reached the maximum at pH=5.5 ((18.1±0.4) U/mL) (Fig. S1b), with a yield of 1140.9 U/g and a productivity of 4.3 U/h (Table S1). The pH values ranging from 5.0 to 6.5 resulted in invertase production varying from 13.0 to 14.0 U/mL. Similar behaviour was observed of *A. niger* IBK1, when invertase production was higher in acidic pH range (4.0-6.0), with maximum invertase production occurring at pH=5.0 (*16*). Dinarvand *et al*. (*24*) evaluated the combined effect of pH-temperature and pH-inoculum on invertase and inulinase production by *A. niger* ATCC 20611. Under these conditions, the maximum invertase production occurred at moderately acidic initial pH=6.5.

The experiments evaluated the effect of agitation speed on *A. carbonarius* PC-4 invertase production, yield and productivity. Four agitation speeds were assayed to evaluate the invertase production by *A. carbonarius* PC-4, maintaining the conditions previously established for 120 h (pH=5.5, 20 °C). The invertase production and additional fermentation parameters increased as the rate of agitation increased up to 180 rpm for 72 h (invertase production of (18.7±0.1) U/mL, yield of 1184.2 U/g and productivity of 4.4 U/h) (Fig. S1c and Table S1). Low agitation speed (130 rpm) promoted the lowest invertase production, reaching the maximum activity of 6.1 U/mL, with a yield of 320.3 U/g and productivity of 0.7 U/h after 48 h.

The influence of agitation speed on enzyme production is an important factor in the fermentation process affecting the successful progress of submerged cultivation in a flask system, since it provides adequate mixing, mass and heat transfer, with consequent improvement of dissolved oxygen levels in the cultivation medium (*26*, *27*). However, the excessive agitation can produce greater mechanical forces and hydrodynamic shear stress, which implies a variety of effects on microbial cell such as rupture of cell wall and change in the morphology of filamentous fungi, variation of efficiency and growth rates besides the formation of undesired products (*27*). Al-Hagar *et al*. (*17*) reported the 1.5-fold increase of invertase production under agitation at 30 rpm compared to static condition, reaching the peak of enzyme production at 150 rpm. In this work, invertase production by *A. carbonarius* PC-4 increased 3-fold when the agitation speed was adjusted from 130 to 180 rpm, with further decrease of enzyme activity when agitation speed was increased to 210 rpm (13.3 U/mL). Dinarvand *et al*. (*28*) related that the increase of agitation speed of shake flask cultivations was followed by a progressive increase of invertase production and cell growth up to 150 rpm although higher agitation rates were deleterious for fungal growth, especially due to the formation of hydrogen peroxide, which is detrimental to the cell.

### Inverted sugar syrup production

**Fig. 2:**
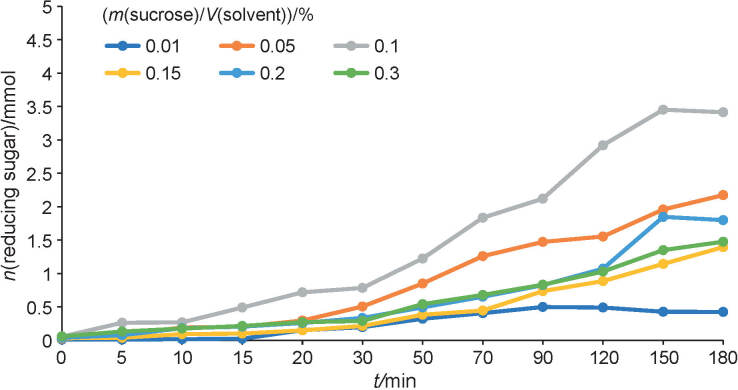
Hydrolysis of sucrose by β-fructofuranosidase from *Aspergillus carbonarius* PC-4 produced under submerged culture

The hydrolysis of sucrose was carried out at initial sucrose concentration 5-30% (*m/V*), 50 °C and pH=5.0. The reactions were initiated with the addition of a total amount of 0.15 U in the reaction system. At low amount of sucrose (1%, *m/V*), the hydrolysis reactions by invertase from *A. carbonarius* PC-4 reached the maximum production of reducing sugars of 498 µmol after 90 min of reaction. By Increasing the sucrose up to 10%, the maximum reducing sugar production was observed after 150 min of reaction (3451.7 µmol) (Fig. 2). Further increase of sucrose content led to decreased hydrolysis rates in the range of 1.9- to 2.4-fold after 180 h.

Keramat *et al*. (*5*) evaluated the effect of sucrose on invertase activity using dynamic light scattering analysis. The authors observed that high sucrose amounts promoted the intensification of sucrose clusters, hindering the enzyme access to the sucrose molecules. Besides this, an increase of sucrose content leads to a reduction of water activity due to changes in the distribution of hydrogen bonds between water and sucrose molecules. Therefore, the decrease of hydrolysis rate by increasing the sucrose amount could also occur due to folded structures of sucrose molecules and their aggregates that remained non-hydrolysed under invertase catalysis.

The invertase synthesized by *A. carbonarius* PC-4 presented two important food industry applications: production of inverted syrup at low sucrose content and the synthesis of fructooligosaccharides at high sucrose content (*9*). The results observed in this study showed that *A. carbonarius* PC-4 invertase is suitable for application in bioprocess development involving initial sugarcane pressing, juice filtration and sugar hydrolysis to obtain high-fructose syrups. Mohd Zain *et al.* (*29*) evaluated the effect of commercial invertases on sucrose hydrolysis of liquid pineapple waste, reaching high concentration of glucose. Additional applications of invertases are preparation of creams and marshmallows, milk powder for infants, candies containing liquefied sugar center, chocolate-covered cherries, digestive aid tablets, artificial honey and plasticizing agents for cosmetics (*30*).

## CONCLUSIONS

Pineapple crown waste for production of β-fructofuranosidase was efficiently used with a newly isolated *Aspergillus carbonarius* PC-4 under submerged conditions. Optimized production of β-fructofuranosidase was achieved using a mixture design (simplex lattice design) and parametric experimental sets leading to 3-fold increase of invertase production from low-cost substrates (pineapple crown and yeast extract). The β-fructofuranosidase produced by *A. carbonarius* PC-4 showed a promising potential for the synthesis of inverted sugars from different initial sucrose contents, which is particularly interesting to the food industry, especially for the production of sweeteners for confectionery and beverage industries. Therefore, the results obtained in this study revealed *A. carbonarius* PC-4 as a new and adapted microbial strain suitable for the synthesis of invertase from an agrowaste of pineapple industry.

## Figures and Tables

**Table S1:** Fermentation parameters of β-fructofuranosidase production by *Aspergillus carbonarius* PC-4 under different cultivation conditions

Fermentation parameter		Invertase activity	
*Y*_p/s_/(U/g)	*r*_p_*/*(U/h)
		Temperature/°C	
15		(599.6±3.8)^c^	(2.23±0.01)^c^
20		(783.2±6.8)^a^	(2.92±0.01)^a^
25		(690.0±5.3)^b^	(2.57±0.01)^b^
30		(407.5±1.0)^d^	(1.52±0.00)^d^
35		(131.1±0.9)^e^	(0.49±0.00)^e^
40		n.d.	n.d.
45		n.d.	n.d.
pH			
3.0		(36.8±1.1)^i^	(0.14±0.00)^h^
3.5		(123.2±4.1)^h^	(0.46±0.00)^g^
4.0		(434.0±5.0)^f^	(1.62±0.01)^e^
4.5		(706.9±10.2)^c^	(2.63±0.01)^c^
5.0		(745.8±8.3)^c^	(2.78±0.01)^c^
5.5		(1149.9±14.3)^a^	(4.28±0.02)^a^
6.0		(934.5±3.1)^b^	(3.48±0.01)^b^
6.5		(906.6±6.1)^b^	(3.37±0.01)^b^
7.0		(639.6±5.1)^d^	(2.38±0.01)^c^
7.5		(573.0±5.06)^e^	(2.13±0.01)^d^
8.0		(307.5±2.04)^g^	(1.14±0.01)^f^
Agitation speed/rpm	*t*/h*		
130	120	320.3±15.0	0.71±0.00
150	60	634.0±24.2	2.83±0.01
180	72	1184.8±19.4	4.41±0.01
210	60	846.9±9.6	3.78±0.02

*Y*_p/s_=yield of invertase, *r*_p_=productivity of invertase, n.d.=not detected. *Time of the highest values of each fermentation parameter
